# Clinical Characteristics of Hypertensive Patients with Obstructive Sleep Apnoea Syndrome Developing Different Types of Left Ventricular Geometry

**DOI:** 10.1155/2021/6631500

**Published:** 2021-01-23

**Authors:** Wojciech Myslinski, Agata Rekas-Wojcik, Andrzej Dybala, Maciej Zakrzewski, Wojciech Barud, Andrzej Prystupa, Grzegorz Dzida, Wiesław Bryl, Jerzy Mosiewicz

**Affiliations:** ^1^Department of Internal Medicine, Medical University of Lublin, Staszica 16, 20-081 Lublin, Poland; ^2^First Military Hospital, Aleje Raclawickie 23, 20-049 Lublin, Poland; ^3^Department of Internal Medicine, Metabolic Disorders and Hypertension, Medical University of Poznan, Szamarzewskiego 84, 60-569 Poznan, Poland

## Abstract

**Objective:**

The objective of the study was to compare polygraphic parameters and selected laboratory parameters in patients with obstructive sleep apnoea (OSA) who develop various types of left ventricular (LV) geometry. *Material and Methods*. The research covered 122 patients with obstructive sleep apnoea and coexisting effectively treated systemic hypertension (95 men, 27 women, average age: 54 ± 10.63). Overnight polygraphy, echocardiography, carotid artery ultrasonography, and laboratory measurements were performed. The patients were classified into four groups, depending on LV geometry. Group 1 comprised patients with normal LV geometry, group 2 included those with LV concentric remodelling. Group 3 and group 4 were patients with LV hypertrophy, concentric or eccentric, respectively.

**Results:**

The most frequent type of LV geometry in the examined population was eccentric hypertrophy (36%). The highest average values of BMI and T-Ch were observed in the group of patients with concentric remodelling (group 2). The most severe respiratory disorders were found in the group of patients developing LV concentric hypertrophy (group 3); however, these differences were not statistically significant in comparison to other groups. Patients with LV eccentric hypertrophy had significantly decreased LV ejection fraction (*p* = 0.0008).

**Conclusions:**

LV eccentric hypertrophy is the most frequent type of LV geometry in OSA patients. Patients with severe sleep-disordered breathing are more likely to develop concentric hypertrophy, while concentric remodelling occurs more frequently among OSA patients with other coexisting conditions, such as obesity or lipid-related disorders.

## 1. Introduction

Obstructive sleep apnoea syndrome (OSA) is a disease which has a significant influence on the cardiovascular system [1]. At apnea-hypopnea index (AHI) ≥15, the prevalence of OSA in the general adult population ranged from 6% to 17%, being as high as 49% in the advanced ages. [2]. Due to high prevalence in the general population, OSA is considered the main cause of secondary systemic hypertension. Other severe cardiovascular complications of OSA include pulmonary hypertension, heart failure, cardiac arrhythmia, and stroke [3]. One of the major organ damages observed in patients with OSA is left ventricular hypertrophy (LVH) which is a significant predictor of total mortality and death due to cardiovascular causes [4, 5]. This negative influence of OSA on the cardiac muscle results from repeated episodes of nocturnal hypoxaemia, leading to increased activity of the sympathetic nervous system [6]. Augmentation of the adrenergic system activity and cyclical changes of pressure within the chest, caused by greater respiratory effort due to the closing of the respiratory tract, increase cardiac afterload, resulting in myocardial hypertrophy. Moreover, cardiac preload is increased as well due to the activation of the renin-angiotensin-aldosterone system (RAAS) [7].

Currently, we distinguish 4 different types of left ventricular (LV) geometry: normal geometry, concentric remodelling, concentric hypertrophy, and eccentric hypertrophy. The highest cardiovascular risk is observed in patients with LV concentric hypertrophy, which typically occurs in people with arterial hypertension or chronic nephropathy [8–10]. At present, numerous studies provide disparate data on the influence of OSA on LV performance. Due to frequent coexistence of OSA with other conditions, such as arterial hypertension, diabetes, or obesity, which also contribute to cardiac remodelling, it is difficult to estimate the actual impact of OSA on LV geometry [11, 12]. The objective of the study was to assess polygraphic parameters and selected laboratory parameters in OSA patients who develop distinct types of LV geometry.

## 2. Material and Methods

### 2.1. Material

The study group comprised 122 patients (95 men, 27 women) aged 23–80 with an established diagnosis of OSA, hospitalised in the Department of Internal Medicine. All patients were effectively treated due to systemic hypertension for at least 6 months prior to the study. Most of the patients (73%) were treated with angiotensin-converting enzyme inhibitors (ACE-I) and angiotensin II receptor blockers (ARB), alone or in combination with diuretics. 36% of the patients were treated with calcium blockers as a monotherapy or in combination with diuretics and/or ACE-I or ARB. Patients with uncontrolled systemic hypertension, atrial fibrillation, chronic heart failure, diabetes mellitus, and impaired renal function (eGFR < 30 ml/min) were not enrolled to the study.

### 2.2. Methods

#### 2.2.1. Biochemical Analysis

Blood samples of all the subjects were collected in the morning before the first meal. Samples of whole blood (5 ml) were collected from the basilic vein into tubes containing ethylenediaminetetraacetic acid tripotassium salt (Sarstedt, S-Monovette with 1.6 mg/ml EDTA-K3) and into tubes with a clot activator (Sarstedt, S-Monovette). Total cholesterol, HDL cholesterol, and LDL cholesterol (T-Chol, HDL-Chol, and LDL-Chol) and triglyceride (TG) concentrations in serum were estimated using routine techniques (COBAS INTEGRA 400 plus analyzer, Roche Diagnostics, Mannheim, Germany). Concentrations were expressed in mg/dl.

Estimated GFR (eGFR) was calculated using Cockroft-Gault formula. Fasting glucose and hsCRP levels were also determined in all patients.

#### 2.2.2. Polygraphy

For or the purposes of OSA diagnosis, polygraphy was performed in all the patients using Sleep Doc Porti 8 apparatus. Sleep staging was done according to the American Academy of Sleep Medicine (AASM) criteria [13].

Apnoea was detected when there was an air flow signal drop by ≥90% from preevent baseline flow, while hypopnoea was scored when there was an air flow signal drop ≥30% from preevent baseline flow. The signal drop should last at least 10 seconds and lead to ≥3% oxygen desaturation from the preevent baseline and/or arousal from sleep. The following polysomnographic parameters were used for further evaluation: Apnoea-Hypopnoea-Index (AHI)—number of apnoea and hypopnoea incidents per hour of sleep; Apnoea Index (AI)—number of apnoea episodes per hour; Hypopnoea Index (HI)—number of hypopnea episodes per hour; Respiratory Disturbance Index (RDI)—defined as a sum of all respiratory disturbances during sleep taking into account apnoea/hypopnoea incidents and awakenings caused by respiratory effort (respiratory effort related arousals—RERA's); Respiratory Disturbance Time Index (RDTI)—hypopnea and apnea duration time per hour; minimal and mean saturation (SaO_2_); t90—a period during which oxygen saturation drops below 90% (SaO_2_ < 90%); Oxygen Desaturation Index (ODI)—number of desaturation (SaO_2_ < 90%) episodes per hour. Obstructive sleep apnoea was diagnosed when AHI was >5 per hour of sleep, with existing clinical symptoms of OSA. OSA severity was estimated according to the guidelines of the American Academy of Sleep Medicine (AASM) on the basis of AHI: mild OSA I (AHI 5–15/h), moderate OSA II (AHI 16–30/h), and severe OSA III (AHI > 30/h) [13, 14].

#### 2.2.3. Echocardiography

Echocardiography was performed in all the patients: M-mode and two-dimensional scans in parasternal long axis view, with a calculation of: interventricular septum (IVS) thickness, LV posterior wall thickness in diastole (PWD), LV end-diastolic diameter (LVED), and LV ejection fraction (EF). On the basis of the data obtained, left ventricular mass (LVM) was calculated. Next, after adjustment to the body surface area (BSA), left ventricular mass index (LVMI) was calculated based on the formula: LVMI = LVM/BSA. Left ventricular relative wall thickness (RWT) was also calculated, defined as a relation of a double value of LV posterior wall thickness to its end-diastolic diameter (2 × PW/LVED). Left ventricular hypertrophy (LVH) was diagnosed in the case of LVMI > 95 g/m^2^ in women and >115 g/m^2^ in men. On the basis of the above-mentioned measurements, the patients were classified into 4 groups of LV geometry. LV concentric remodelling was defined as normal LV mass and RWT ≥ 0.42, concentric hypertrophy as RWT ≥ 0.42 and LVMI > 115 g/m^2^ in men or LVMI > 95 g/m^2^ in women, while eccentric hypertrophy as RWT < 0.42 and LVMI > 115 g/m^2^ in men or LVMI > 95 g/m^2^ in women [15].

#### 2.2.4. Carotid Artery Ultrasonography

Additionally, in order to assess the presence of atherosclerosis, intima-media thickness (IMT) of the common carotid artery was assessed by using Vivid 4 ultrasound system, equipped with 7-10 MHz transducer. The average value of 3 measurements of IMT was used for statistical analysis.

## 3. Statistical Analysis

Statistical calculations were made with STATISTICA 10.0 software. Quantitative variables were expressed as average values and standard deviations. Due to the lack of normal distribution of variables, nonparametric tests were used in the calculations (ANOVA on ranks, Kruskal-Wallis test). The statistical analysis for qualitative variables was performed on the basis of a chi-squared test. Parameter correlations were checked by calculating Pearson's *R* correlation coefficient. All statistical tests were carried out at 95% statistical significance level (alpha 0.05), with *p* < 0.05 regarded as statistically significant.

## 4. Results

**Figure 1:**
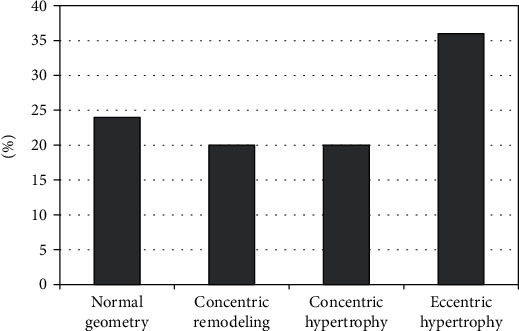
Distribution of LV geometry types in patients with OSA.

The study group consisted of 122 patients of whom 95 (77.9%) were men; the average age of the patients was 53.55 ± 10.63. The patients were classified into four groups depending on LV geometry type: group 1: normal geometry with 29 (24%) patients (22 men; average age: 55.45 ± 9.98), group 2: concentric remodelling with 24 (20%) patients (21 men; average age: 49.83 ± 11.28), group 3: concentric hypertrophy with 25 (20%) patients (20 men; average age: 56.32 ± 9.32), and group 4: eccentric hypertrophy with 44 (36%) patients (32 men; average age: 52.75 ± 11.03). The most frequent type of LV geometry among patients with obstructive sleep apnoea was eccentric hypertrophy (36%, group 4). Distribution of LV geometry types is presented in Figure 1.

The statistical analysis did not show significant differences in the examined polygraphic and laboratory parameters among groups of OSA patients developing different types of LV geometry. However, we demonstrated that the patients with features of concentric hypertrophy had more severe sleep-disordered breathing (the highest values of AHI, AI, RDI, and RDTI) in comparison to other groups, even though the differences described were not statistically significant. In patients with LV concentric hypertrophy, the average value of minimal SpO_2_ was 68.24 ± 16.28 and was insignificantly lower compared to patients developing concentric remodelling and eccentric hypertrophy (71.58 ± 12.58 and 74.75 ± 10.35, respectively). Also, in subjects with LV concentric hypertrophy, mean values of CRP were insignificantly increased in comparison to subjects with concentric remodelling and eccentric hypertrophy. On the other hand, the group with concentric remodelling included patients with the highest values of BMI, HI, HR, T-Ch, and the lowest HDL. In comparison to other groups, there were statistically significant differences in ejection fraction in the group of patients with eccentric hypertrophy, which was the lowest in group 4 (*p* = 0.000813).

All measured anthropometric, polygraphic, and laboratory parameters are listed in Table 1.

**Figure 2:**
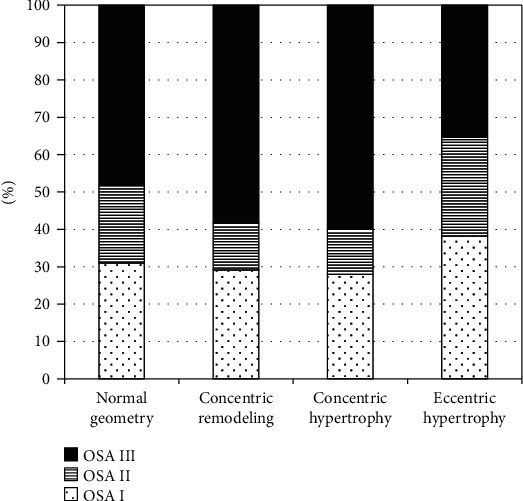
Severity of OSA in groups with different types of LV geometry.

In patients developing LV concentric hypertrophy and LV concentric remodelling, the prevalence of severe OSA was observed. The distribution of patients with different severity of OSA is presented in Figure 2.

Furthermore, Pearson's correlation was analysed for all the examined groups of patients, and it was observed that there was a significant negative correlation of t90 with LVMI (*R* = −0.2505; *p* = 0.044). The results are shown in Table 2.

## 5. Discussion

Respiratory disorders during sleep, and especially obstructive sleep apnoea, play a significant role in initiation and progression of cardiovascular diseases. This is associated primarily with the influence of sleep apnoea on changes in the structure of the heart and vessels, which is largely due to excessive activation of the sympathetic nervous system, triggered by episodic hypoxaemia [6]. One of the elements of a pathological mosaic of structural changes in the cardiovascular system of OSA patients is the development of LVH [16–19]. The main risk factors of LVH in the general population are age, male gender, obesity, and arterial hypertension [20]. The literature gives contradictory data on the influence of OSA itself on LVH, demonstrating prevalence of both concentric or eccentric LVH among OSA patients [11, 16, 21]. Damy et al. demonstrated that AHI, independently of systemic hypertension and BMI, was a factor associated with LVH [17]. By contrast, Noda et al. showed that, despite significant correlations of AHI and duration of SaO_2_ < 90% with LVMI, this index also significantly correlated with BMI and blood pressure [11]. Similar results were obtained by Niroumand et al. who, in a large group of patients, demonstrated that LVH in OSA patients is mainly connected with coexisting obesity, arterial hypertension, and age [12].

In the majority of studies which assessed the influence of OSA on the heart structure, LVH was generally defined as an increase in LV mass, disregarding the fact that the left ventricle develops various types of geometry in reaction to pathological factors, such as arterial hypertension or obesity. LV abnormal geometry includes concentric remodelling and two distinct types of hypertrophy: concentric and eccentric. Population studies demonstrate that the highest cardiovascular risk is posed to patients with features of concentric hypertrophy, which is a typical response of the myocardium to increased left ventricular afterload, connected primarily with arterial hypertension [22]. This has been confirmed by numerous studies, for example, those conducted by Cuspidi et al., who compared patients with resistant hypertension and with hypertension well-controlled by medication [9]. They showed that concentric hypertrophy was dominant in the group of patients with resistant hypertension, while eccentric hypertrophy predominated in the group of patients with well-controlled hypertension. Similarly, in the Resist-POL study, conducted in a population of patients with resistant hypertension, concentric hypertrophy was the most frequent type of LV disorder [23]. It was also demonstrated that the independent factors contributing to the incidence of concentric hypertrophy in this group of patients were age, OSA (AHI > 15/h) and systolic pressure at night. Moreover, a positive correlation was shown between AHI and LVMI, and between AHI and RWT (*r* = 0.282, *p* = 0.001 and *r* = 0.335, *p* = 0.0001) [19].

In the authors' own research, it was demonstrated that the most frequent type of LV geometry observed in OSA patients was eccentric hypertrophy, whereas other authors found that concentric hypertrophy was dominant in patients with respiratory disorders during sleep [16]. The objective of present study was therefore to assess potential differences in polygraphic and laboratory parameters among OSA patients who developed various types of LV geometry.

The majority of the patients investigated in our study were men, and the groups of patients with various types of LV geometry were not significantly different in terms of age and BMI. The potential influence of arterial hypertension was difficult to assess, which resulted firstly from the impossibility to exactly specify the duration of hypertension, and secondly, from various therapies of hypertension throughout the years. That is why the impact of a “therapeutic” factor cannot be unambiguously defined. However, it should be emphasised that the patients eligible for the study had arterial hypertension efficiently treated with various therapeutic schemes.

In our present study, the most frequently observed type of LV geometry among the patients was eccentric hypertrophy (36%). In this group (group 4), EF values were the lowest in comparison to other groups. Similarly, Damy et al. in their study of patients with respiratory disorders during sleep, noted greater prevalence of eccentric hypertrophy when systolic LV dysfunction coexisted [17]. Drager et al., in turn, observed more frequent occurrence of concentric hypertrophy among patients with OSA but without systolic failure [18].

The most severe OSA was observed in a group of patients with LV concentric hypertrophy. These differences, noticeable albeit statistically insignificant, were expressed in the lowest values of minimal saturation and the highest values of AHI, AI, RDI, and RDTI in comparison to other groups. Moreover, in this group, there was also the highest share of patients with the most severe respiratory disorders during sleep. Furthermore, the patients with concentric hypertrophy had the highest average CRP that can be caused by recurrent episodes of hypoxaemia, which, as described in the literature, results in endothelial dysfunction and formation of free radicals of oxygen, responsible for induction of inflammatory reaction [24, 25].

One of the polysomnographic parameters describing severity of OSA is desaturation time (t90). It is a period during which saturation drops below 90%. In Pearson's analysis, carried out for the whole examined population, we demonstrated a statistically significant negative correlation between LVMI and t90. These results could suggest that there is no influence of the desaturation period on induction of myocardial hypertrophy. However, it should be emphasised that the severity of respiratory disorders during sleep is determined by a number of parameters, such as AHI, AI, or a period of desaturation below 90%. While defining OSA severity only on the basis of a total number of apnoea and hypopnoea incidents, we often forget that not only quantity but also “quality” of apnoea episodes determine the severity of the disease. Thus, at a comparable period of desaturation below 90%, a determinant of OSA severity is not duration, but extent of desaturation. In other words, two episodes of apnoea lasting 20 seconds with a saturation drop to 85% will probably cause lesser neurohumoral disturbances than one 40-second episode of apnoea with desaturation to 65%. Such deep apnoea episodes, resulting in a significant drop in saturation, can be a factor stimulating secretion of vasoactive substances to a much larger degree than in patients with moderate desaturation. In the population examined in our study, we observed the lowest values of minimum saturation in patients with LV concentric hypertrophy. These results can indicate that the patients in this group more frequently experienced episodes of deeper desaturation. In research on rats, it was demonstrated that long-term hypoxia is connected with an increase in IL-6 and with LV eccentric hypertrophy, whereas short and interrupted episodes of hypoxia are associated with a higher level of TNF-alpha and with the presence of concentric hypertrophy [26].

LV hypertrophy is typical finding in patients with chronic kidney disease (CKD). Patients with concentric left ventricular hypertrophy had decreased eGFR in comparison to patients developing LV concentric remodelling and eccentric hypertrophy, but these differences were not statistically significant and probably are not responsible for the development of LV concentric hypertrophy. It has been demonstrated that OSA is associated with glomerular hyperfiltration and may be an independent predictor of proteinuria [27]. The role of sympathetic and renin-angiotensin-aldosterone system activation seems to be crucial in OSA patients. In our unpublished study, we found that patients with mild and severe OSA had significantly increased eGFR in comparison to age-matched control group. Further studies should be performed to assess the impact of potential renal dysfunction on the LV performance in patients with OSA.

There are increasing number of evidences that alterations in the circadian blood pressure profile in nondipper hypertensive patients promote the development of cardiovascular changes including vascular remodelling and left ventricular hypertrophy. Recurrent episodes of hypoxemia stimulate the release of proinflammatory cytokines, and increased stimulation of chemoreceptors activates the sympathetic and renin-angiotensin-aldosterone systems [28]. Aldosterone is a recognized factor in stimulating cardiac hypertrophy and myocardial fibrosis. In our study, we did not assess neurohumoral disturbances in patients with sleep-disordered breathing. It should be emphasized, however, that in patients with LV concentric hypertrophy, the strong risk factor of cardiovascular events, we did not demonstrate increased thickness of carotid artery intima-media. This may indicate that in patients with apnoea, the combination of haemodynamic and neurohumoral disorders leads mainly to dysfunction of the heart muscle and to a lesser extent has a negative effect on the vascular system.

## 6. Conclusions

The literature gives contradictory data on the influence of sleep apnoea on cardiac remodelling. Due to frequent coexistence of conditions which could potentially be responsible for cardiac remodelling, it is difficult to unambiguously indicate the main determinant of the development of distinct types of LV remodelling. The results of our research indicate that OSA patients most often develop eccentric hypertrophy independently of increased body mass, whereas patients with more intense respiratory disorders during sleep typically develop concentric hypertrophy. These findings are consistent with studies of patients with arterial hypertension, where it was demonstrated that concentric hypertrophy was associated with the highest risk of cardiovascular complications. It therefore seems important to continue the research aimed at identifying of multiple factors, including neurohumoral ones, which in the population of OSA patients determine the development of different types of LV geometry, which can contribute to better cardiovascular risk stratification in this group of patients.

## Figures and Tables

**Table 1:** Average values of the measured parameters in patients with obstructive sleep apnoea with different types of LV geometry.

Types of LV geometry	Normal geometry	Concentric remodelling	Concentric hypertrophy	Eccentric hypertrophy	*p*
Variable	Mean ± SD	Mean ± SD	Mean ± SD	Mean ± SD	
Age (years)	55.45 ± 9.98	49.83 ± 11.28	56.32 ± 9.32	52.75 ± 11.03	NS
BMI (kg/m^2^)	33.49 ± 6.26	33.70 ± 5.05	33.09 ± 5.28	31.81 ± 4.34	NS
AHI (h^−1^)	33.39 ± 24.92	36.65 ± 23.54	40.43 ± 23.63	33.58 ± 24.27	NS
AI (h^−1^)	24.26 ± 24.23	26.02 ± 23.02	31.34 ± 24.32	23.18 ± 21.28	NS
HI (h^−1^)	9.12 ± 9.02	10.58 ± 6.80	8.92 ± 8.14	9.87 ± 7.81	NS
RDI (h^−1^)	83.26 ± 72.72	88.06 ± 65.53	112.68 ± 83.78	83.60 ± 73.17	NS
RDTI (min/h)	13.80 ± 12.69	14.94 ± 11.32	18.93 ± 14.00	14.07 ± 11.40	NS
Mean SpO_2_ (%)	89.62 ± 4.04	89.63 ± 3.12	89.72 ± 4.75	90.68 ± 3.30	NS
Minimal SpO_2_ (%)	73.17 ± 10.33	71.58 ± 12.58	68.24 ± 16.28	74.75 ± 10.35	NS
t90 (min)	26.07 ± 28.27	23.29 ± 28.03	21.76 ± 24.95	17.23 ± 20.29	NS
ODI (h^−1^)	36.71 ± 27.39	41.46 ± 26.52	46.24 ± 28.30	40.65 ± 26.71	NS
Heart rate (mean)	61.00 ± 7.93	65.83 ± 11.16	61.64 ± 6.54	61.50 ± 8.73	NS
eGFR (ml/min)	97.61 ± 17.12	103.22 ± 20.04	92.48 ± 16.65	100.41 ± 19.94	NS
hsCRP (mg/l)	4.30 ± 4.93	2.98 ± 2.33	5.16 ± 9.37	3.22 ± 5.22	NS
T-Ch (mg/dl)	195.50 ± 44.48	199.55 ± 57.78	187.44 ± 49.18	184.54 ± 39.79	NS
Ch-LDL (mg/dl)	133.27 ± 64.16	126.00 ± 41.59	103.70 ± 44.79	116.94 ± 57.55	NS
Ch-HDL (mg/dl)	42.51 ± 11.20	38.41 ± 8.21	44.33 ± 10.12	42.59 ± 11.66	NS
TG (mg/dl)	195.96 ± 106.92	175.65 ± 64.09	170.10 ± 86.37	181.94 ± 110.16	NS
Glycaemia (mg/dl)	108.61 ± 26.38	100.74 ± 18.17	98.59 ± 16.60	102.11 ± 27.19	NS
EF (%)	64.18 ± 5.70	67.14 ± 6.40	65.29 ± 7.04	59.79^∗^ ± 8.65	<0.05
IMT (mm)	0.9 ± 0.22	0.88 ± 0.18	0.91 ± 0.19	0.91 ± 0.21	NS

Values are expressed as mean ± SD. ^∗^*p* < 0.05.

**Table 2:** Pearson's correlations between LVMI and polygraphic measurements.

	LVMI (g/m^2^)	
	*R*	*p*
AHI	0.0296	0.815
AI	0.0518	0.682
HI	-0.1257	0.318
RDI	0.0926	0.463
RDTI	0.1021	0.418
SpO_2_ mean	0.1961	0.117
SpO_2_ min	0.0446	0.724
t90	-0.2505	0.044^∗^
ODI	-0.0088	0.944

*R*: Pearson's correlation coefficient. ^∗^*p* < 0.05.

## Data Availability

Database including all collected maesurements are available from the corresponding author if needed.
